# Reduction of pluripotent gene expression in murine embryonic stem cells exposed to mechanical loading or Cyclo RGD peptide

**DOI:** 10.1186/s12860-017-0148-6

**Published:** 2017-11-14

**Authors:** Olesja Hazenbiller, Neil A. Duncan, Roman J. Krawetz

**Affiliations:** 1 McCaig Institute for Bone and Joint Health, University of Calgary, Calgary, AB Canada; 2 Department of Civil Engineering Schulich School of Engineering, University of Calgary, Calgary, Canada; 3 Department of Cell Biology and Anatomy, Cumming School of Medicine, University of Calgary, 3330 Hospital Drive N.W, Calgary, AB T2N 4N1 Canada

**Keywords:** Embryonic stem cell, Collagen type I, Cyclic RGD peptide, Confined compression, Integrins, Mechano-transduction

## Abstract

**Background:**

Self-renewal and differentiation of embryonic stem cells (ESCs) is directed by biological and/or physical cues that regulate multiple signaling cascades. We have previously shown that mESCs seeded in a type I collagen matrix demonstrate a loss of pluripotent marker expression and differentiate towards an osteogenic lineage. In this study, we examined if this effect was mediated in part through Arginylglycylaspartic acid (RGD) dependent integrin activity and/or mechano-transduction.

**Results:**

The results from this study suggest that mESC interaction with the local microenvironment through RGD dependent integrins play a role in the regulation of mESC core transcription factors (TF), Oct-4, Sox 2 and Nanog. Disruption of this interaction with a cyclic RGD peptide (cRGDfC) was sufficient to mimic the effect of a mechanical stimulus in terms of pluripotent gene expression, specifically, we observed that supplementation with cRGDfC, or mechanical stimulus, significantly influenced mESC pluripotency by down-regulating core transcription factors. Moreover, our results indicated that the presence of the cRGDfC peptide inhibited integrin expression and up-regulated early lineage markers (mesoderm and ectoderm) in a Leukemia inhibitory factor (LIF) dependent manner. When cRGDfC treated mESCs were injected in Severe combined immunodeficiency (SCID) mice, no tissue growth and/or teratoma formation was observed, suggesting that the process of mESC tumor formation in vivo is potentially dependent on integrin interaction.

**Conclusions:**

Overall, the disruption of cell-integrin interaction via cRGDfC peptide can mimic the effect of mechanical stimulation on mESC pluripotency gene expression and also inhibit the tumorigenic potential of mESCs in vivo.

## Background

Embryonic stem cell (ESCs) functions can be controlled by their surrounding microenvironment. Recent research by our group and others has shown that physical factors, such as stiffness of the extracellular matrix (ECM) and the mode of mechanical stimulus can provide appropriate cues to trigger cell responses, e.g. self-renewal and differentiation [1–4]. However, the challenge remains to identify the underlying mechanism of how physical factors direct cell fate decisions.

In the field of mechano-transduction, growing interest is directed toward integrins and their role in converting mechanical signals into an appropriate biochemical response. Integrins are transmembrane proteins composed of an alpha/βeta domain and act as mechanical link between the ECM and the intracellular cytoskeleton network. In addition to cell adhesion, integrins can mediate signal transduction events and influence cell functions such as differentiation, proliferation, survival and apoptosis [5, 6]. To date, 24 integrin constellations (18 alpha and 8 βeta) have been identified, subdivided into four groups: RGD, collagen, leukocyte, and laminin receptors, based on their recognition sequences in the matrix [5, 7].

RGD dependent integrins (αvβ3, α5β1, αvβ5, etc.), recognize the RGD (Arg-Gly-Asp) amino acid sequence found in proteins such as fibronectin, vitronectin, and fibrinogen when RGD is accessible: i.e. through RGD immobilization to non-binding matrices [3–5]. Although all RGD dependent proteins recognize the RGD amino acid sequence, the selectivity and affinity of an integrin to this sequence depends on amino acid structure (i.e., linear versus cyclic form) [7]. For example, cyclo (Arg-Gly-Asp-d-Phe-Cys) (cRGDfC) possesses high affinity to αvβ3 integrin [8].

Collagen receptors (α1β1, α2β1, α10β1, α11β1, etc.) are considered as RGD independent integrins but have been shown to partially bind RGD if accessible in the collagen matrix. For example, on thermally or proteolytic denatured collagen matrix, and during tissue repair and regeneration [9–11]. Subsequently, when this cryptic RGD motif becomes accessible in the collagen matrix, RGD dependent integrins can recognize and bind to it.

In this study, we evaluated the role of RGD dependent integrins in mESCs when seeded in a collagen matrix. Previously our group has shown, that when mESCs are seeded in collagen type I matrix (mESC-Col I), these constructs can contribute to bone regeneration in vivo without forming tumors [4, 12]. It has been speculated that cyclic loads during the healing process reduced the expression of pluripotent markers in mESCs, and thus inhibited tumorigenesis, which is supported by the findings of two groups. Nakajima et al. [13] showed that incorporation of undifferentiated ESC in an immobilized knee joint resulted in tumor formation while in a mobilized joint they contributed to cartilage formation. The group of Lynch et al. [14] found that metastatic breast cancer cells injected in mice tibia models can inhibit osteolysis and tumor formation under axial compressive load while bone degradation occurred without load.

To distinguish between the mechanical and biochemical effects in vivo, we have previously undertaken a study to identify and reproduce the mechanical environment in vivo within the transplanted mESC-Col I construct in vitro. In that study, we observed that a biologically relevant mechanical stimulus reduced the gene expression of pluripotent markers (Oct 4, Nanog, Sox 2, Rex 1), as well as the oncogene ERas. However, the signaling mechanism involved in regulating the cells remained unknown. Therefore, in the current study, we have investigated if integrins may play a role in the mechanical regulation of mESCs, specifically, if RGD dependent integrins in combination with mechanical stimulus can regulate mESC pluripotent gene expression.

There is a strong rationale to suggest that the interaction of cells with RGD dependent integrins, such αvβ3 integrin, has a role in regulating the pluripotency of mESCs. For example, during development, αvβ3 is required for embryo implantation in mouse and human [15], and during bone resorption, the interaction of αvβ3 with osteopontin and bone sialprotein is essential [16]. It has also been shown that numerous cell types require αvβ3 to transduce mechanical signals [17, 18], however, little is known about the role of RGD dependent integrins in mESCs. Therefore, we hypothesized that during cyclic loading integrin binding sites become accessible in the collagen matrix and the interaction of mESCs with RGD dependent integrins would contribute to the down-regulation of pluripotent transcription mechanisms.

## Methods

### Cell culture

Murine ESC-D3 (ATCC, Manassas, VA) were cultured on 0.1% gelatin (Sigma, St. Louis, MO) coated T-75 flasks (Sigma, St. Louis, MO) in high glucose Dulbecco’s Modified Eagle Medium (DMEM, Invitrogen, Carlsbad, CA) supplemented with 15% fetal bovine serum (FBS, Invitrogen, Carlsbad, CA), 1% non-essential amino acid (NEAA), 10,000 U/mL of penicillin and 10,000 μg/mL streptomycin, and 0.1 mM ß-Mercaptoethanol (ßME) (all Invitrogen, Carlsbad, CA). To maintain mESC pluripotency, mESCs were cultured in the presence of 1000 U/mL leukemia inhibitory factor (LIF, Chemicon, Billerica, MA) and passaged upon reaching confluence every third day. Cells were maintained in a humidified incubator with 5% CO_2_ at 37 °C.

### Preparation of mESC-col I constructs in the presence and absence of cRGDfC peptide

Murine ESC were embedded in a type I collagen matrix as previously described [10]. Briefly, mESCs at passage 8 to 10 were trypsinized and re-suspended in β-glycerol phosphate medium (βGP, Sigma, St. Louis, MO). βGP medium consisted of high glucose 5 x DMEM powder medium supplemented with 10 mM βGP and with FBS, NEAA, penicillin-stereptomycin solution, and ßME equal to the standard cell culture procedure. To prepare one collagen construct, 1,000,000 cells were re-suspended in 200 μL βGP medium and mixed with 800 μL of bovine collagen type I solution (3 mg/mL) (PureCol®, Advanced Biomatrix, Poway, CA) and of 20 μL sodium hydroxide. For the integrin studies, cRGDfC (cyclo (Arg-Gly-Asp-d-Phe-Cys)) peptide (Peptides international Inc. Louisville, KY) was dissolved in 3% citric acid and this was supplemented to mESC-Col I solution to achieve a final concentration of 0.5 mM cRGDfC. Finally, mESC-Col I solution, in the presence and absence of cRGDfC peptide, was pipetted into a 12 well plate and allowed to polymerize in a humidified incubator with 5% CO_2_ at 37 °C.

### Effect of cRGDfC peptide in loaded and unloaded mESC-col I constructs

The mESC-Col I constructs were subjected to confined compressive load using a modified Flexcell FX-4000™ system as described [2, 19]. Briefly, mESC-Col I constructs were prepared as stated above and allowed to polymerize for 24 h. Samples were then transferred to the compression culture plate, the loading system was assembled and constructs pre-loaded using a tare load of 0.5 N for 5 min. Finally, mESC-Col I constructs were compressed to 3.5% strain at 1 Hz for 40 h (two cycles of 4 h loading followed by 16 h of rest [2, 19]).

Four groups were assessed for mESC gene expression: 1) non-loaded mESC-Col I constructs, 2) non-loaded mESC-Col I constructs in the presence of 0.5 mM cRGDfC, 3) loaded mESC-Col I constructs, and 4) loaded mESC-Col I constructs in the presence of 0.5 mM cRGDfC. After completion of loading, loaded and unloaded samples were collected in 1.5 mL Eppendorf tubes and frozen at −80 °C until RNA extraction.

### Effect of cRGDfC peptide on mESCs without collagen matrix

Murine ESCs were seeded at a cell density of 30,000 cells/well in a 0.1% gelatin coated 12 well plate. Cells were supplemented with 500 μL complete DMEM and allowed to attach in the presence of 1000 U/mL LIF for 24 h. The media was then changed and mESCs cultured in the presence of 0.5 mM cRGDfC peptide for 24 h. Murine ESCs cultured in the presence or absence of LIF and without cRGDfC served as controls. Cell morphology was evaluated using a Nikon TS 100 (Nikon Instruments Inc. Melville, NY) after which samples were collected for viability and gene expression analysis.

### Cell viability

For 2D studies, viability was assessed using trypan blue (Sigma, St. Louis, MO) exclusion in duplicates using TC10™ Automated Cell Counter (BioRad, Mississauga, ON) and for 3D studies using LIVE/DEAD Viability Kit (Invitrogen, Carlsbad, CA) as previously described [2, 19]. Images were obtained from mESC-Col I constructs in the presence and absence of cRGDfC peptide in duplicates at three random locations using a Zeiss LSM 510 confocal microscope and percentage of cell viability was calculated using NIH Image J (v1.44p) by dividing green fluorescent cells by the sum of red and green fluorescent cells.

### Gene expression

RNA was extract from mESCs in static culture and from mESC-Col I using an established Trizol protocol (Invitrogen, Carlsbad, CA) and reverse transcribed into complementary DNA using High Capacity cDNA Reverse Transcription Kit (Applied Biosystems, Carlsbad, CA) with a total RNA input of 2 μg per 50 μL reaction. Quantitative real time polymerase chain reaction (qrt-PCR) was performed on pluripotent markers (Oct-4, Rex 1, Sox 2 and Nanog), integrin subunits (αv, β3, α1, and β1), E-Cadherin and tri-lineage markers (Brachyury, EOMES, Nestin, Otx2, Sox 7, Gata 6) gene using TaqMan® Universal PCR Master Mix with no AmpErase (Applied Biosystems, Carlsbad, CA).

### In vivo tumor formation assay

Fox Chase CB-17 severe combined immunodeficiency (SCID) mice were obtained from Charles River and housed in the single-barrier animal facility of the Faculty of Medicine, University of Calgary. Animal protocols were carried out as approved by the Animal Care Committee at the University of Calgary according to the standards of the Canadian Council of Animal Care. Mice were fed ad libitum with a standard diet and water. One million viable mESCs were injected into the skin fold of the inner thigh of 4 mice per group. Undifferentiated cells were injected as single cells, whereas cell-loaded collagen constructs where injected intact. Each animal was injected in both thighs with the same treatment group. The animals were sacrificed (anesthetized followed by cervical dislocation) at 30 days post mESC injection. Emerging tissue material was dissected. Excised tissues were fixed overnight in 4% PFA at 4 °C and then embedded in paraffin. Sections were stained in hematoxylin/eosin according to standard procedures.

### Flow Cytometry

The cells were dissociated and resuspended in 500 μl of 90% MeOH and left for 5–10 min at room temperature. The cells were then centrifuged, the liquid was removed and 500 μl of 0.1% Tween 20 was added to permeabilize the cells for 20 min at room temperature. The cells were centrifuged again, the liquid was removed, and 50 μl of Tween buffer and 0.5 μg of antibody was added to each tube and incubated in the dark for 30–45 min at room temperature. All antibodies were purchased from Ebioscience either directly conjagted to Alexa 488 or FITC; or if unconjugated, were directly conjugated to Dylight 488 (Abcam). The cells were then washed three times with FACs buffer then resuspended in FACs buffer. The cells were then measured using an LSRII flow cytometer (BD Biosciences). The results were analyzed using FlowJo software.

### Statistical analysis

The fold changes in gene expression were calculated using the established 2^-∆∆CT^ method by normalizing against the 18S housekeeping gene across all samples and then calculating the relative fold changes against mESC static control cells (per experimental replicate). Significant differences between groups were statistically evaluated using one-way ANOVA and Tukey’s multiple-comparison test using GraphPad Prism 6 software (GraphPad Software Inc., USA). The significance level was set to 0.05.The gene expression changes were plotted as mean ± standard deviation (SD). At least 3 independent experimental replicates with 3 technical replicates per experiment were carried out per experiment (unless stated otherwise).

## Results

### Effect of cRGDfC in loaded and unloaded mESC-col I constructs

**Fig. 1:**
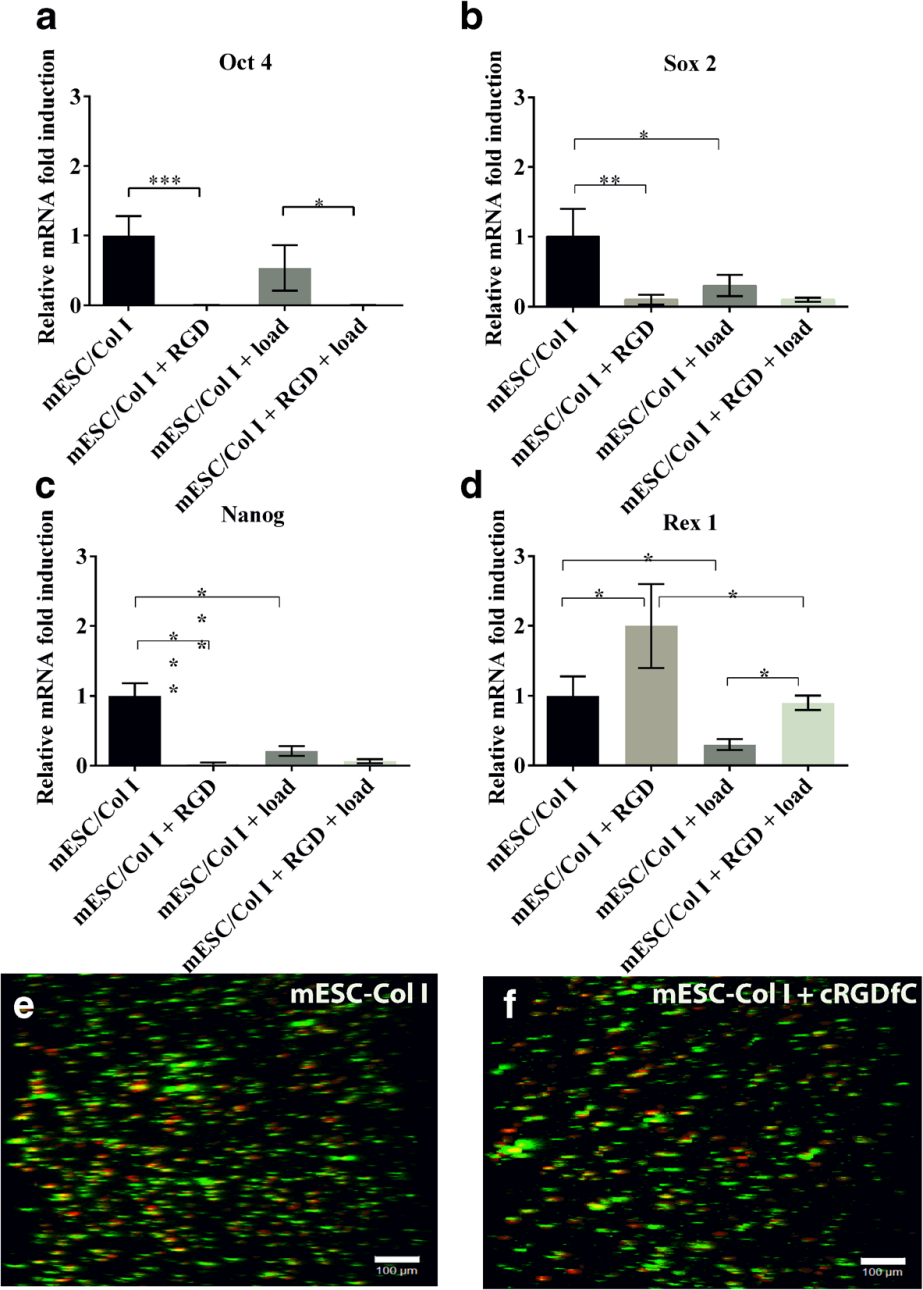
The presence of cRGDfC peptide with and without compression down-regulated mESC pluripotent markers such as (**a**) Oct 4, (**b**) Sox 2 and (**c**) Nanog, while (**d**) Rex 1 gene expression was up-regulated. Application of compressive force on mESC-Col I constructs with free integrin receptor resulted in the down-regulation of all tested pluripotent markers. Mean ± SD, *n* = 3. (***;*p* < 0.0001; **;*p* < 0.001; *;*p* < 0.05). Z-stack images showing that cell viability in the presence and absence of cRGDfC was not significantly different (**e**) mESC-Col I, 83% ± 7 and (**f**) mESC-Col I + cRGDfC, 75.3% ± 2.5, Scale bar = 100 μm

The presence of cRGDfC peptide significantly influenced the gene expression of mESCs seeded in collagen matrix (mESC-Col I) in comparison to mESC-Col I constructs with free integrin receptor. Specifically, the transcription factors Oct 4, Sox 2 and Nanog (Fig. 1 a, b, c) were down-regulated in the presence of cRGDfC peptide. Oct 4 and Nanog were down-regulated 99% and 97.5%, respectively, while the expression of Sox 2 was down-regulated 90%. However, the presence of cRGDfC peptide had the opposite effect on Rex1 gene expression, which was doubled.

Since we have previously demonstrated a link between pluripotency and mechano-transduction in mESCs without or without Col I supplementation, we next sought to examine the role of RGD dependent integrins in the regulation of pluripotency related gene expression under mechanical stimulation (e.g. loading). mESC-Col I constructs were cyclically compressed to 3.5%, at 1 Hz for 40 h. Without cRGDfC (mechanical loading alone), the gene expression of pluripotent markers was down-regulated (Oct 4–50%; Sox 2–70%; Nanog - 80%; and Rex 1–70%) in comparison to mESC-Col I with no load. When cRGDfC peptide was present with load, a similar gene expression pattern was observed compared to unloaded mESC-Col I constructs with cRGDfC (Fig. 1 a, b, c, d).

Interestingly, and as observed before, Rex 1 showed the opposite response compared to Oct 4, Sox 2 and Nanog. In the presence of cRGDfC peptide, Rex 1 gene expression was up-regulated in both loaded and unloaded constructs (Fig. 1).

In order to verify that the changes in gene expression were not due to cell death, cell viability throughout the entire construct was determined in the presence and absence of cRGDfC in unloaded constructs. Untreated mESC-Col I samples demonstrated a viability of 83% ± 7 while cell viability in presence of cRGDfC peptide was 75.3% ± 2.5 (Fig. 1 e, f). Additionally, as observed previously, loading had no effect on viability of the cells.

### Effect of cRGDfC peptide on selective integrin subunits in loaded and unloaded mESC-col I constructs

**Fig. 2:**
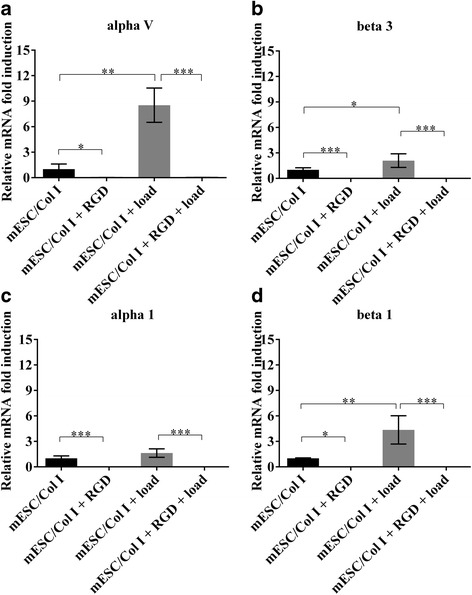
Relative mRNA gene expression of RGD dependent integrin subunits (**a**, **b**) (alpha V and beta 3) and collagen dependent integrin subunits (**c**, **d**) (alpha1 and beta1) in response to the presence of cRGDfC peptide and/or compression. Mean ± SD, n = 3 independent experimental replicates. (***;p < 0.0001; **;p < 0.001; *;p < 0.05)

Since it has been previously demonstrated that integrin activation can result in a positive or negative feedback loop [20], we next evaluated whether the presence of cRGDfC peptide was able to regulate the gene expression of selective RGD and collagen dependent integrin subunits αvβ3 and α1β1. The supplementation of cRGDfC to mESC-Col I constructs completely suppressed the gene expression of all tested integrin subunits. Interestingly, even α1, that is partially RGD independent, was negatively regulated by cRGDfC treatment (Fig. 2 a-d). While mechanical load and cRGDfC treatment both down-regulated the expression of pluripotency related genes (except Rex 1), in response to mechanical load, the expression of αv (750%), β3 (110%) and β1 (350%) subunits were significantly up-regulated, while α1 expression was not significantly affected by mechanical load. However, when cRGDfC peptide was supplemented to mESC-Col I in constructs and subjected to load, the effects of load on integrin expression on the mESC-Col I constructs were negated with the addition of cRGDfC peptide (Fig. 2 a-d). Interestingly, cRGDfC peptide treatment not only influenced the gene expression of RGD dependent integrin subunits (αv, β3), but also that of collagen dependent (α1, β1) subunits.

### Effect of cRGDfC peptide on mESCs in the absence of a collagen matrix

Based on the ability of the cRGDfC peptide to regulate both pluripotent and integrin gene expression of mESCs within Col I constructs, it was next determined to investigate if the peptide was able to regulate mESC gene expression, morphology or cell viability in static culture with and without LIF.

**Fig. 3:**
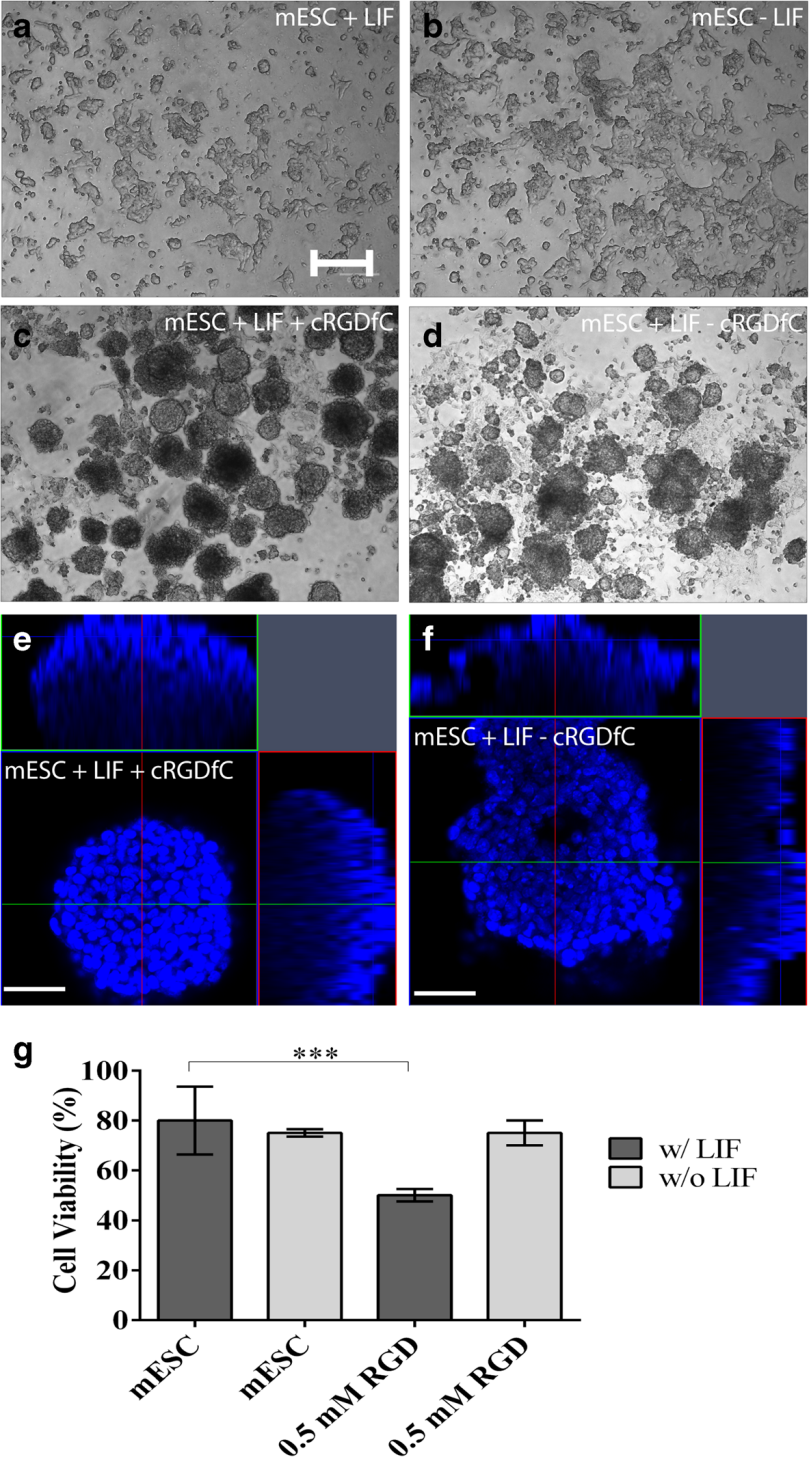
Effect of cRGDfC peptide on mESC morphology and viability when cultured in a dish without the presence of collagen matrix. (**a**) mESC with LIF and (**b**) mESC without LIF remained attached and showed no visible differences in cell morphology; (**c**) mESC with LIF + cRGDfC and (**d**) mESC without LIF + cRGDfC formed round shaped aggregates and detached from the dish. Scale bar, 200 μm. The morphology of aggregates formed in the presence (**e**) or absence (**f**) of LIF was also examined using DAPI staining with 3D confocal imaging and reconstruction. Scale bar, 100 μm. (**g**) Cell viability significantly decreased in the presence of LIF and cRGDfC to 50% in comparison to mESC w/LIF

It has been well characterized that removal of LIF will initiate spontaneous differentiation of mESCs, with a change in morphology occurring 2–4 days after LIF removal. In line with these previous studies, no obvious differences in the morphology of mESCs was observed 24 h after LIF removal (Fig. 3 a, b). However, in the presence of cRGDfC and LIF, mESCs formed 3D floating cell aggregates which were similar to mESC morphology found in suspension cultlure [5] (Fig. 3 c). In the absence of LIF, cRGDfC treatment also led to the formation of aggregates and detachment from the surface, however, the aggregates no longer demonstrated a uniform appearance and morphology (Fig. 3 d). To achieve a greater understanding on the difference in morphology of mESCs in the presence of cRGDfC with or without LIF, aggregates were isolated and examined using confocal microscopy (Fig. 3 e, f). In the presence of cRGDfC and LIF, the cells within mESC aggregates are tightly packed and the overall aggregate is of uniform geometry, however, when LIF is removed, the cells within the aggregate appear to be less uniform with empty space present throughout. Additionally, the overall geometry of the aggregates are less uniform (Fig. 3 f). Interestingly, we also observed a decrease in cell viability with the addition of cRGDfC, however, this was only observed in the presence of LIF (Fig. 3 g).

**Fig. 4:**
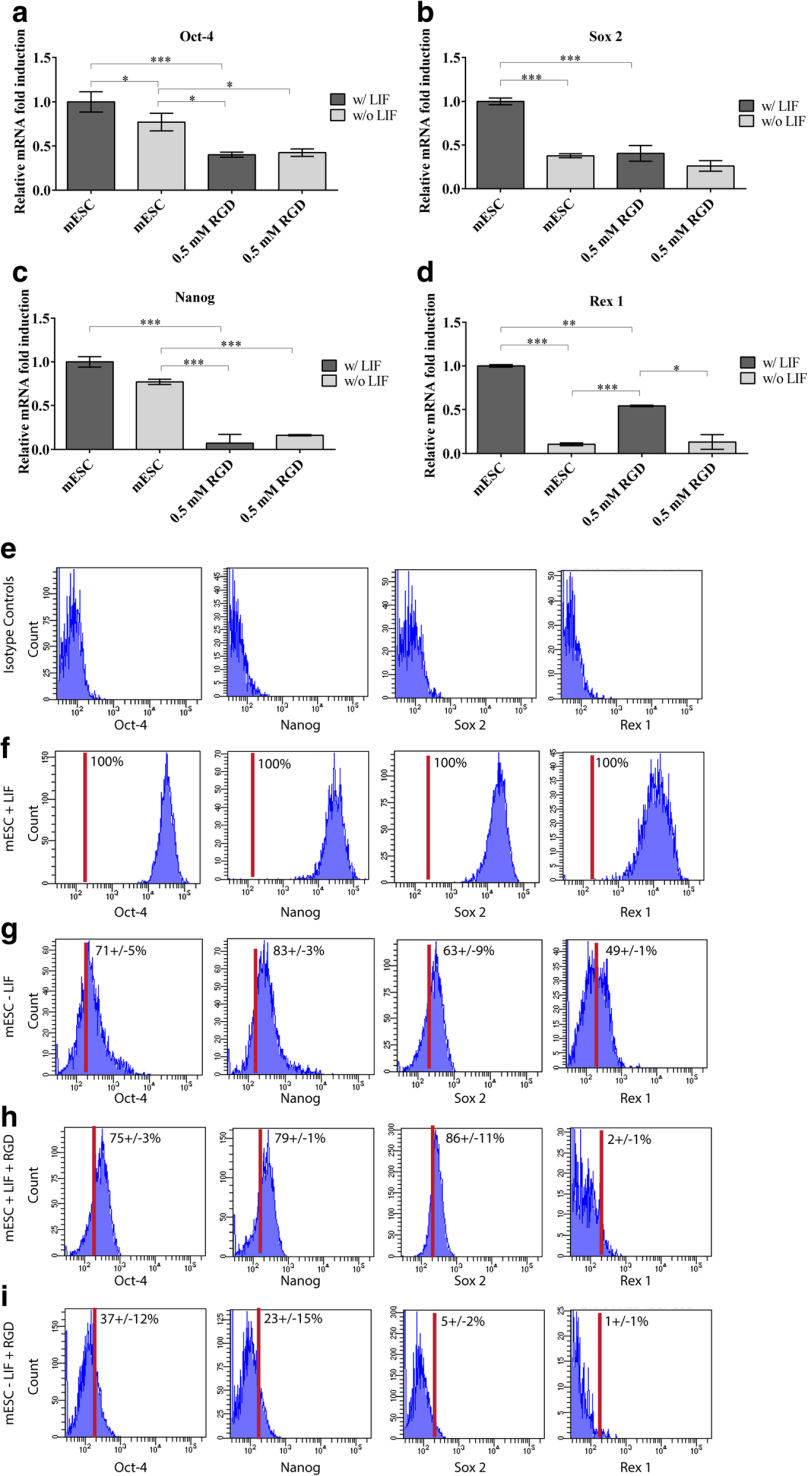
Relative mRNA gene expression of pluripotent markers in the presence of cRGDfC, w/ and w/o LIF (**a**-**d**). Supplementation of 0.5 mM cRGDfC peptide down-regulated mESC pluripotency in the presence of LIF. Mean ± SD, n = 3 independent experimental replicates. (***;p < 0.0001; **;p < 0.001; *;p < 0.05). Flow cytometry validation of PCR data (**e**-**i**). Representative data from 3 independent experimental replicates

Since we observed that the addition of cRGDfC to mESC static cultures supplemented with LIF caused the cells to form aggregates in suspension similar to when mESCs are cultured in suspension bioreactors, we were interested in determining if this also regulated the expression of pluripotent genes as is the case in suspension bioreactors. As expected, upon removal of LIF, all tested pluripotent markers were down-regulated with the greatest reduction observed for Rex 1 (Fig. 4 a-d). Similar to mESCs in collagen matrices, the supplementation of cRGDfC peptide plus LIF had a similar effect to removal of LIF on gene expression. The gene expression of Oct-4, Sox 2 and Nanog was significantly down-regulated 60%, 60% and 90%, respectively, in comparison to mESC cultured in the presence of LIF. Contrasted to what was observed in collagen matrices, Rex 1 was significantly downregulated in the presence of cRGDfC peptide (Fig. 4 d). The combined effect of LIF removal and supplementation of cRGDfC peptide had no effect on the expression Oct 4, Sox 2 and Nanog, but the levels of Rex 1 were further decreased in comparison to mESC cultured in the presence of cRGDfC and LIF.

These mRNA results were validated at the single cell protein level using flow cytometry. Isotype controls demonstrated limited non-specific staining for all antibodies tested (Fig. 4 e). One hundred percent of mESC cultured in media containing LIF expressed Oct-4, Nanog, Sox 2 and Rex 1 (Fig. 4 f), while as expected the percentage of cells expressing pluripotent markers decreased after LIF removal (Fig. 4 g). When mESCs were cultured in the presence of LIF and cRGDfC peptide, the marker expression resembled mESCs culture without cRGDfC – LIF except for Rex 1 staining, which was almost completely absent from the population (Fig. 4 h). When the mESCs were culture without LIF but with cRGDfC, cells expressing pluripotent markers were severely decreased compared to all other treatment groups (Fig. 4 i).

### Effect of cRGDfC peptide on mESC tumorigenic potential in vivo

**Fig. 5:**
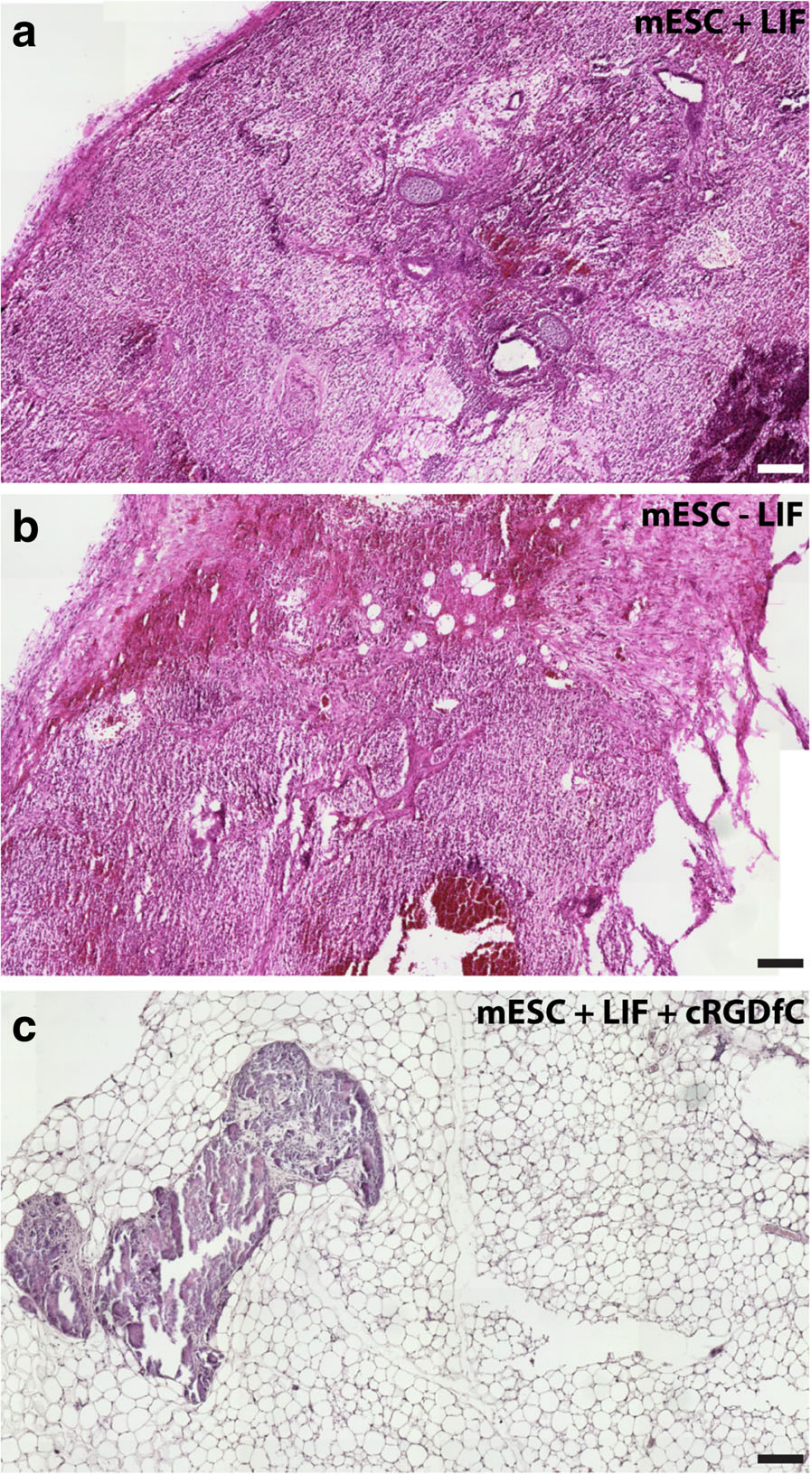
Teratoma formation in SCID mice. Undifferentiated mESCs cultured in the presence of LIF generated teratomas in all (4/4) mice injected (**a**). mESCs cultured in the absence LIF for 24 h still produced teratomas in all (4/4) mice injected (**b**). When cRGDfC was supplemented to mESCs cultured in the presence of LIF, no teratomas (0/4) were observed, however one mouse (1/4) did present with a growth containing fat tissue (**c**). In the absence of LIF, but in the presence of cRGDfC, no teratomas (0/4) or tissue growths of any type were observed. Scale bar, 200 μm

Since it was observed that cRGDfC could reduce the expression of pluripotent genes in the presence or absence of LIF in statically cultured mESCs, it was next tested if cRGDfC treated cells still could produce tumors when injected in SCID mice. As expected, mESCs cultured in the presence of LIF generated teratomas in all (4/4) mice injected (Fig. 5 a). When LIF was removed and the mESCs were cultured for an additional 24 h, teratomas were still observed in all (4/4) mice injected (Fig. 5 b). Interestingly, however, when cRGDfC was added to mESCs cultured in the presence of LIF for 24 h and then injected into mice, no teratomas (0/4) were observed but one mouse (1/4) did present with a growth containing mainly fat tissue (Fig. 5 c). When LIF was removed from mESCs and cRGDfC was added in its place 24 h prior to injection, no teratomas (0/4) or tissue growths of any type were observed.

### Effect of cRGDfC peptide on mESC surface receptors without collagen matrix

**Fig. 6:**
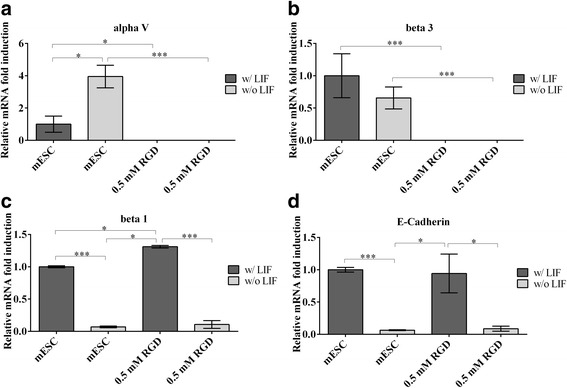
Relative mRNA gene expression of cell surface receptors in the presence of cRGDfC, w/ and w/o LIF. Supplementation of cRGDfC shut down the expression of RGD dependent integrin subunits, (**a**) alphaV and (**b**) beta 3, independent of LIF. The expression of (**c**) beta 1 and (**d**) E-Cadherin appeared to be LIF dependent. In the presence of LIF beta 1 and E-Cadherin expression was up-regulated and without LIF down-regulated. Mean ± SD, n = 3 independent experimental replicates. (***;p < 0.0001; **;p < 0.001; *;p < 0.05)

To examine the potential mechanism behind the lack of tumors in vivo after mESC treatment with cRGDfC, mESCs were cultured in the presence of LIF in static culture and the expression of integrins and cadherins known to play a role in pluripotency and tumorigenesis were examined [21–23]. In the presence of LIF, cells expressed αv, β3 and β1 integrin subunits, and E-Cadherin (Fig. 6 a-d). Upon removal of LIF, the expression of αv was up-regulated 300%, while the β3 submit remained unchanged and the integrin subunit β1 and E-Cadherin were both down-regulated 90% (Fig. 6 a-d). The supplementation of cRGDfC peptide completely inhibited the expression of αv and β3, irrespective of the presence of LIF, whereas the effect of cRGDfC on β1 integrin subunit appeared to be LIF dependent, while cRGDfC did not appear to differentially regulate the expression of E-cadherin in the presence or absence of LIF.

### Effect of cRGDfC peptide on mESC lineage specification

**Fig. 7:**
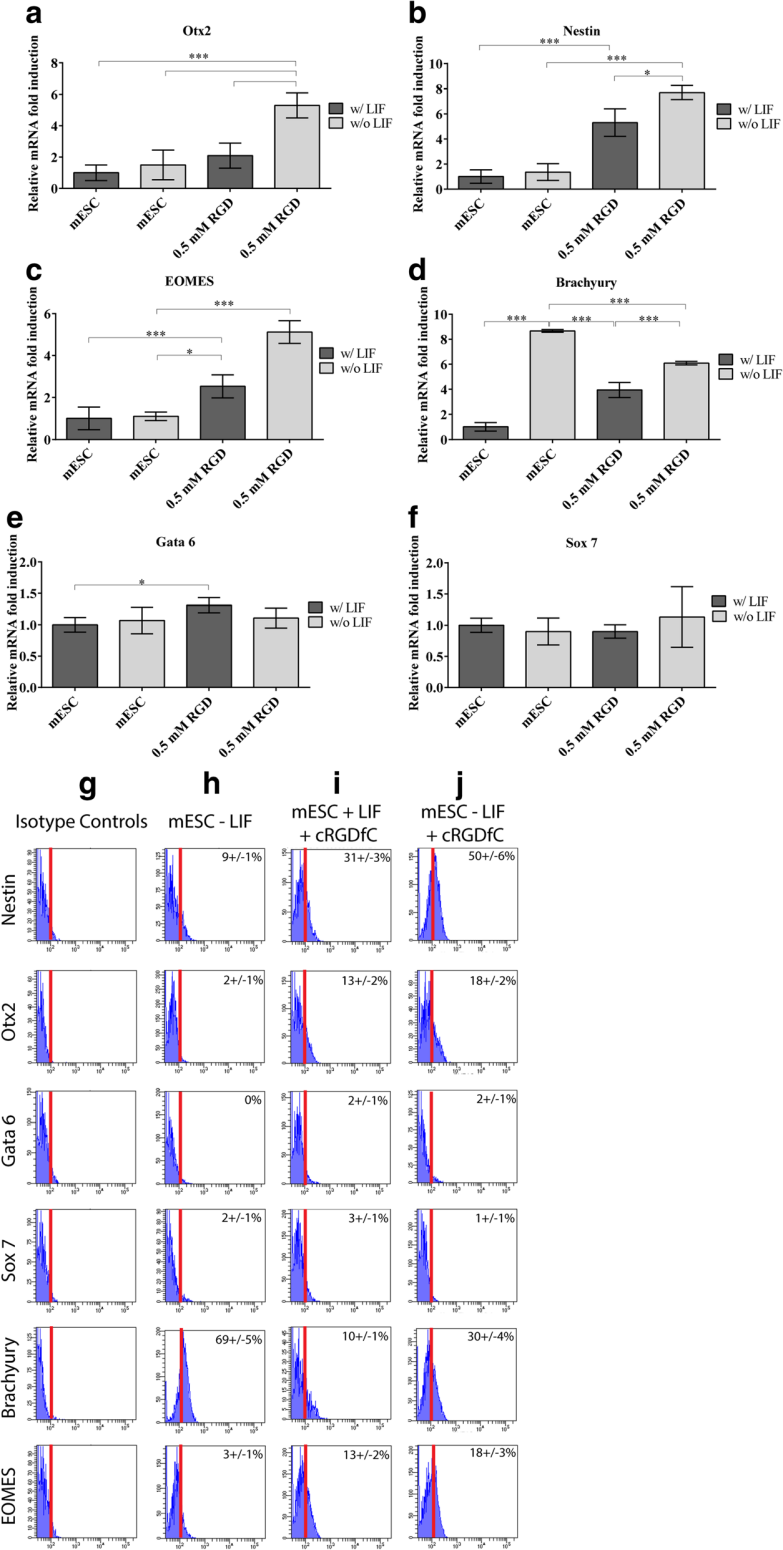
Tri-lineage marker analysis in the presence of cRGDfC, w/ and w/o LIF. Early ectodermal (**a**,**b**), mesodermal (**c**,**d**) and endodermal (**e**,**f**) markers were examined within the different treatment groups by quantitative PCR. (***;p < 0.0001; **;p < 0.001; *;p < 0.05). Flow cytometry validation of PCR data (**e**-**i**). Representative data from 3 independent experimental replicates. These results were confirmed using flow cytometry in the Oct-4 negative cell population of each treatment group (**g**-**j**)

Since it could be possible that the mESCs might be undergoing lineage specification/commitment in response to the removal of LIF and/or the supplementation of cRGDfC peptide resulting in reduced tumorigenesis, tri-lineage markers were examined at the mRNA and single cell protein (flow cytometry level). Two early ectoderm (Nestin, Otx2), mesoderm (Brachyury, EOMES) and endoderm (Gata 6, Sox 7) were analyzed. At the mRNA expression level, the ectodermal marker, Otx2 was only significantly upregulated in the absence of LIF plus cRGDfC peptide (Fig. 7 a), while Nestin was upregulated in both conditions (LIF +/−) with cRGDfC peptide present (Fig. 7 b). The mesoderm marker EOMES was upregulated in both conditions (LIF +/−) with cRGDfC peptide present (Fig. 7 c), while Brachyury was also upregulated in both conditions (LIF +/−) with cRGDfC peptide, but also in mESCs without LIF and without cRGDfC peptide (Fig. 7 d). For the early endodermal marker Gata 6, a minor upregulation was observed in the presence of cRGDfC peptide plus LIF (Fig. 7 e) and no upregulation of Sox 7 was observed across any treatment group (Fig. 7 f). To confirm mRNA expression data, a flow cytometry strategy was employed with appropriate isotype controls (Fig. 7 g). Initially two distinct populations of each marker were observed (positive and negative)(Data not shown), and after investigation, it was observed that nearly all Oct-4 positive cells (in each treatment group) were negative for early lineage specification markers (Data not shown). Therefore, analysis was performed for each marker set (endoderm, ectoderm, mesoderm) on the Oct-4 negative population in each treatment group. Since no Oct-4 negative cells were present in mESCs cultured with LIF, no further analysis was conducted on this treatment group. In mESC cultured without LIF, few to no ectoderm (Nestin, Otx2) or endoderm (Gata 6, Sox 7) marker positive cells were observed, however, over half the cell population began to express Brachyury, but not EOMES (Fig. 7 h). In the presence of cRGDfC peptide plus LIF, Nestin and Otx2 positive cells were observed (Fig. 7 i), while no Gata 6 or Sox 7 positive cells were observed (Fig. 7 i). Approx. 10–12% of the cell in the presence of cRGDfC peptide plus LIF expressed Brachyury and EOMES (Fig. 7 i). When LIF was removed in the presence of cRGDfC peptide, approx. Half of the cells expressed Nestin, while 17% expressed Otx2 (Fig. 7 j). Again, few to no cells expressed Gata 6 or Sox 7 (Fig. 7 j), and approx. 31% of the population expressed Brachyury, while 17% expressed EOMES (Fig. 7 j).

## Discussion

Numerous external (i.e., mechanical or biological) and internal (i.e., signaling through integrins and cytokines) stimuli have been previously shown to regulate stem cell fate decision by initiating a cascade of intracellular signalling events. When cells experience external mechanical stimulation such as compression, one key mechanism of the cellular response is through mechanosensitive cell surface receptors such as integrins [24]. Integrins link the extracellular environment to the cell interior and it has been demonstrated that even small disruptions in integrin-ECM interaction can regulate gene expression and cell behaviour [25, 26]. Therefore, it has been be observed that the addition of non-mobilized peptide sequences to cell-ECM constructs can reduce the cell-ECM interaction by occupying integrin receptors and thus limit mechano-transduction events via integrins [27]. Our previous studies examining the behaviour of mESC embedded in Col I gels demonstrated a reduction of pluripotent gene express and a repression of tumorigenesis after implantation into SCID mice regardless if the cell constructs were implanted immediately or after an incubation period [4]. Therefore, the goal of the present study was to evaluate the effect of non-mobilized cRGDfC peptide presence on the gene expression of pluripotent markers (Oct 4, Nanog, Sox 2, Rex 1) and integrin subunits in loaded and unloaded mESC-Col I constructs to determine if disrupting the integrin-ECM interaction of mESCs was sufficient or necessary for the previous observations and/or could the peptide treatment mimic the effect of Col I supplementation in regards to decreasing pluripotent gene expression and tumorigenic potential of mESCs. In this study, we observed that the presence of a cRGDfC peptide (e.g. integrin-ECM disruption) played a role in the regulation of mESC core transcription factors, Oct-4, Sox 2 and Nanog when seeded in a 3D Col I matrix. Interestingly, all observations made in within the collagen matrices in regards to pluripotent gene expression were not applicable to Rex 1, as this transcription factor acted oppositely to the core transcription factors. One possible explanation is that the cRGDfC and/or mechanical stimuli did not induce the cells to terminally differentiate during the time points examined. It has been suggested the Rex 1 expression is tightly linked to the undifferentiated state and may not be directly linked to potency [28], therefore, the reason that we did not see a reduction in Rex 1 expression, but did see a reduction in Oct-4, Sox 2 and Nanog was that the cRGDfC and/or mechanical stimuli was reducing the potency of ESCs, but not inducing differentiation at the timepoint examined. Another possible explanation is that Rex 1 may be more of a ‘naïve’ ESC marker vs. a ‘primed’ ESC marker [29] and in this study we did not employ methodology to distinguish naïve vs. primed ESCs. When mESCs were cultured without collagen matrices however, Rex 1 acted in a similar manner as the other core pluripotency markers when LIF was removed and/or cRGDfC peptide added, suggesting that Rex 1 expression may be more mechanosensitive specifically in an integrin-dependent manner, however, this hypothesis would require further study.

Previously, in 2D culture systems, undifferentiated mESC (D3 cell line) have been shown to express a variety of RGD (αv, α5, β1) and laminin dependent (α3, α6, α7, β1) integrin subunits but not that of collagen dependent subunits (α1, α2) when grown on different substrates including gelatin and Col I [30]. Similar to our results, in prior studies, mESC (D3 cell line) grown on gelatin expressed high levels of integrin subunits forming the RGD dependent integrin complex (αvβ3) but the expression of alpha subunits (α1, α2), which form integrin receptors to Col I ECM, were missing. When mESC were seeded in a 3D collagen matrix and initiated an osteogenic differentiation [2, 4], the integrin expression pattern changed through a switch from RGD (αvβ3) to collagen receptor (α1β1). Additionally and similar to other studies, our results in the current study indicate that RGD receptors may play an essential role in regulated mESC pluripotency, while it is thought that collagen receptors have a more developmental role [30, 31]. That being said, a limitation of the current study was that it was solely undertaken with D3 mESCs, while these are a commonly used mESC cell line, it is possible that differential results might be observed in mESCs derived from different strains, therefore, we caution that these results may not be generalizable to all ESCs without further study.

As RGD dependent integrins bind only partially to the collagen matrix, one might expect that the presence of cRGDfC would have no effect on mESC fate and that signal transduction is predominantly mediated by collagen receptors. However, when cRGDfC peptide was added to mESC-Col I constructs, pluripotent markers were down-regulated, accompanied by a complete loss of αvβ3 and α1β1 collagen receptor expression. It has been shown that when integrins bind to RGD sequence, integrin clustering can take place and the recruitment of other integrins and adhesion molecules can occur [32]. This clustering effect might not have occurred in the mESC-Col I constructs since we observed a decrease in collagen receptor expression. However, since we only examined the expression at the transcript level, it would require further testing to confirm this hypothesis.

The initial purpose of our study was to better understand integrin mediated mechano-transduction in mESC-Col I constructs, however, the supplementation of cRGDfC peptide showed no effect on the expression of pluripotent markers in response to load and that the presence of cRGDfC alone was sufficient to achieve a down-regulation of the core pluripotency transcription factors, which is a similar result to mechanical loading alone. These observations led us to investigate the mechanism underlying RGD dependent integrins in regulating mESC pluripotency at an early stage of differentiation, and by removing the surrounding collagen matrix we evaluated the effect of cRGDfC in the presence and absence of LIF. Cells cultured in the presence of LIF and cRGDfC peptide formed round 3D aggregates similar to aggregates found during the formation of early germ layer tissues (e.g. extraembryonic endoderm, neuro-spheres, pelleted mesodermal cultures). ESCs grown in suspension culture or embroid body formation, all present with clear delineated borders [33–35]. Interestingly, the formation of this packed cell aggregates appear to be LIF dependent, since mESCs grown without LIF but with cRGDfC peptide, generated poorly formed aggregates with ill-defined borders. However, in both conditions (LIF +/−), αv, β3 and α1 integrin subunits were inhibited while β1 and E-Cadherin were significantly up-regulated. Our results suggest that blocking RGD dependent cell-ECM interaction can stimulate cell-cell contact formation potentially via E-Cadherin and in future studies it would be interesting to determine if this treatment is regulating fate specification in these cells over a longer time period. However, we did examine early markers of tri-lineage specification and observed that within the Oct-4 positive population, little to no lineage specification markers were expressed, however, in the Oct-4 negative population within each treatment group it appears that early fate specifications may have begun and this also appeared to be effected by the cRGDfC peptide. Specifically, it appeared that the removal of LIF induced specification into a mesodermal lineage (expression of Brachyury and EOMES), however, in the presence of cRGDfC peptide the presence of cells positive for the ectodermal markers Nestin and Otx2 were increased within the population, while the percentage of mesodermal positive cells was decreased. The cRGDfC peptide appeared to have little effect on endodermal lineage specification. While it has been previously demonstrated that a number of neuronal cell types express αvβ3 integrins including radial glia cells [36, 37], the role of RGD dependent integrins in ectodermal/neuronal differentiation has been suggested, but is less clear [38]. It is important to highlight, however, that the fold increases in gene expression of the tri-lineage markers were quite low and for the flow cytometry analysis the sub-population of positive cells were only identifiable in the Oct-4 negative population of the cells. Since it has been previously shown that some of these markers can be transient and given the short duration of the cRGDfC peptide treatment, in our opinion, this does not provide conclusive results that the cRGDfC peptide induced terminal differentiation/specification of the cells, but instead appears to promote some level of expression of early ectodermal/neuronal markers, however, this would need to be investigated in more depth with long term differentiation including molecular and functional outcome measures to verify this observation.

Similar to what we observed when mESCs were encapsulated in a Col I matrix, we demonstrated that during both conditions (LIF +/−) in 2D cultures, mESCs, expression of core pluripotent transcription factors were regulated by the cRGDfC. Therefore, we can speculate that the observed down-regulation of mESC pluripotency was not a direct effect of LIF signaling through the JAK-STAT pathway, but rather by blocking of RGD dependent cell-ECM interaction. It is also important to highlight that we observed constant up-regulation of Rex1 in the presence of cRGDfC, when surrounded by collagen matrix, while the core transcription factors, Oct-4, Sox 2 and Nanog were down-regulated. The functional role of the core transcription factors on pluripotency and embryogenesis has been extensively reviewed and reported by others [39–43], however, as stated earlier the functional role of Rex1 is not as clear. Although Rex 1 is a recognized marker for mESC pluripotent state, it has also been found that Rex1 function is not required to maintain mESC pluripotency and its role lies more in influencing cell differentiation, cell cycle regulation, and cancer progression [44, 45].

Taken together, we have demonstrated that cRGDfC peptide treatment can regulate the gene expression of mESC core transcription factors, and that manipulation of RGD dependent integrins can mimic the effects of mechanical loading by down-regulating the expression of pluripotent markers. While further studies are required to directly assign this role to a specific RGD dependent integrin through molecular approaches (e.g. knockouts, knock-downs), but based on previous literature, it is likely that αvβ3 integrin plays a role in the regulation of mESC pluripotency since the affinity and selectivity of cRGDfC to αvβ3 has been previously demonstrated [8, 46].

## Conclusion

In conclusion, we have shown that RGD dependent integrins play a critical role in the ability of mESCs to interact with their local microenvironment and to respond to mechano-transduction. Furthermore, disruption of cell-ECM connection through RGD inhibition reduced mESC pluripotency in a 3D environment. Additionally, cRGDfC treatment of static mESCs in the presence or absence of LIF was able to inhibit tumor formation upon transplantation of mESCs into mice, suggesting that the interaction of the mESCs within ECM molecules in vivo is at least partly required for tumorigenesis. Lastly, we have demonstrated that cRGDfC peptide treatment of mESCs induces the expression of early ectodermal lineage markers in a sub-set of cells. The findings of this study demonstrate the importance of RGD dependent integrins in mESC and provide new insights of mechano-transduction mechanisms in mESC.

## Abbreviations

cDNA: complementary DNA
Col I: type I Collagen
cRGDfC: Cyclic RGD peptide
DMEM: Dulbecco’s Modified Eagle Medium
ECM: Extracellular matrix
ESC: Embryonic Stem cell
FBS: Fetal bovine serum
LIF: Leukemia inhibitory factor
qrt-PCR: Quantitative real time polymerase chain reaction
RGD: Arginylglycylaspartic acid
RNA: ribonucleic acid
SCID: Severe combined immunodeficiency
ßME: ß-Mercaptoethanol
TF: Transcription Factor
